# Transcriptional host–pathogen responses of *Pseudogymnoascus destructans* and three species of bats with white-nose syndrome

**DOI:** 10.1080/21505594.2020.1768018

**Published:** 2020-06-17

**Authors:** Christina M. Davy, Michael E. Donaldson, Hana Bandouchova, Ana M. Breit, Nicole A.S. Dorville, Yvonne A. Dzal, Veronika Kovacova, Emma L. Kunkel, Natália Martínková, Kaleigh J.O. Norquay, James E. Paterson, Jan Zukal, Jiri Pikula, Craig K.R. Willis, Christopher J. Kyle

**Affiliations:** a Environmental and Life Sciences Program, Trent University, Peterborough, Canada; b Wildlife Research and Monitoring Section, Ontario Ministry of Natural Resources and Forestry, Peterborough, Canada; c Department of Ecology and Diseases of Game, Fish and Bees, University of Veterinary and Pharmaceutical Sciences Brno, Brno, Czech Republic; d Department of Biology and Centre for Forest Interdisciplinary Research (C-FIR), University of Winnipeg, Winnipeg, Canada; e Institute of Vertebrate Biology, Czech Academy of Sciences, Brno, Czech Republic; f Natural Resources DNA Profiling and Forensics Centre, Trent University, Peterborough, Canada

**Keywords:** Disease ecology, emerging infectious diseases, *Eptesicus fuscus*, host–pathogen interactions, susceptibility, *Myotis lucifugus*, *Myotis myotis*, *Pseudogymnoascus destructans*, virulence

## Abstract

Understanding how context (e.g., host species, environmental conditions) drives disease susceptibility is an essential goal of disease ecology. We hypothesized that in bat white-nose syndrome (WNS), species-specific host–pathogen interactions may partly explain varying disease outcomes among host species. We characterized bat and pathogen transcriptomes in paired samples of lesion-positive and lesion-negative wing tissue from bats infected with *Pseudogymnoascus destructans* in three parallel experiments. The first two experiments analyzed samples collected from the susceptible Nearctic *Myotis lucifugus* and the less-susceptible Nearctic *Eptesicus fuscus*, following experimental infection and hibernation in captivity under controlled conditions. The third experiment applied the same analyses to paired samples from infected, free-ranging *Myotis myotis*, a less susceptible, Palearctic species, following natural infection and hibernation (n = 8 sample pairs/species). Gene expression by *P. destructans* was similar among the three host species despite varying environmental conditions among the three experiments and was similar within each host species between saprophytic contexts (superficial growth on wings) and pathogenic contexts (growth in lesions on the same wings). In contrast, we observed qualitative variation in host response: *M. lucifugus* and *M. myotis* exhibited systemic responses to infection, while *E. fuscus* up-regulated a remarkably localized response. Our results suggest potential phylogenetic determinants of response to WNS and can inform further studies of context-dependent host–pathogen interactions.

## Introduction

Survival in a host–pathogen system depends on the ability of the pathogen to exploit resources of its host, and the ability of the host to protect itself from the pathogen. This arm race of adaptations can escalate to the extinction of the host or the pathogen [1–3]. Understanding host–pathogen interactions and the drivers of susceptibility is required to track the spread of novel pathogens, to mitigate impacts of emerging pathogens, and to identify factors driving spill-over of zoonotic diseases. These efforts can be complicated when host–pathogen interactions and disease outcomes shift among different contexts, including host species, stages of infection, or environmental conditions [4–6].

In the bat white-nose syndrome (WNS) system, bats are infected during hibernation when the psychrophilic fungus *P seudogymnoascus destructans* invades the skin, causing high mortality in naïve populations of susceptible species [1,7]. Infection with *P. destructans* triggers a physiological cascade in susceptible species that includes a fever response [8,9], increased arousal frequency [10,11,12], and depletion of fat stores [13,14]. After hibernation, recovering bats may exhibit immune response inflammatory syndrome and associated wing damage [15]. Lesions (cupping erosions packed with fungal hyphae) on the wings may also increase evaporative water loss [13].

Palearctic bat species that have co-evolved with *P. destructans* develop only mild clinical signs [16–18], while susceptibility to WNS varies among Nearctic species that were first exposed since *P. destructans* was introduced to eastern North America [7,19,20]. Understanding the genetic and behavioral basis for interspecific variation in susceptibility to WNS has been a critical research focus since the disease was described [7,14,17,21–27]. However, our understanding of the molecular response of hibernating bats to WNS is largely limited to studies of the little brown bat [*Myotis lucifugus;* 8,28,29]. A previous attempt to contrast transcriptomic response of susceptible and tolerant bat species to WNS was inconclusive because the tolerant species (Palearctic *Myotis myotis)* did not develop clinical signs of the disease [21], while a study of euthermic, free-ranging *M. myotis* did not detect any transcriptomic responses to infection with *P. destructans* [30].

Characterizing interspecific variation in the molecular responses to WNS during hibernation could identify drivers of susceptibility, tolerance, and resistance to fungal infections [8,24,28] and inform studies of other fungal pathogens. It could also improve predictions of the effects of WNS as *P. destructans* continues to spread [17,25,31,32]. Specifically, the accuracy of such predictions could be improved by understanding whether the molecular response of hibernating bats to *P. destructans* reflects evolutionary history (i.e., is more similar among related species) or reflects a species’ susceptibility, tolerance, or resistance to the pathogen [24,33]. Finally, comparing the molecular response of *P. destructans* to growth on different species of bat could identify virulence factors produced most consistently by the fungus, which could be targeted for treatment.

Once *P. destructans* invades the epidermis it begins to produce riboflavin, causing lesions to fluoresce under ultraviolet (UV) light and contributing to the necrosis observed in WNS-affected bats following arousal [34,35]. Fungal loads are higher in lesion-positive wing tissue from hibernating bats, compared to lesion-negative wing tissue from the same individuals [8,34,36], suggesting that fungal growth accelerates following the invasion of the integumentary structures. *Pseudogymnoascus destructans* does not appear to secrete keratinases but does secrete lipases [29,34,37], which may facilitate the digestion of sebum and keratin allowing invasion of deeper tissues of the wing. Collagen digestion may then be mediated by the secretion of proteases such as Destructin-1, −2, and −3 [37–39]. However, potential targeted responses of *P. destructans* to saprophytic and pathogenic growth on bat wings have not yet been characterized. Two competing hypotheses exist: (1) that *P. destructans* is an “accidental pathogen” whose saprophytic growth also facilitates invasion of bat tissue, and (2) that *P. destructans* exhibits specific pathogenic responses in the wing tissue of bats, which differ from its saprophytic growth *in vitro*, on cave substrates, or on the surface of bat wings [37].

Inter-individual variation in host responses to disease complicates comparative studies of host–pathogen interactions [40]. In bats with WNS, individual and interspecific variation can be untangled by collecting paired samples of wing tissue from infected individuals, which do or do not contain lesions [8,17,33,34,36]. These lesion-positive and lesion-negative samples can then be used to compare fungal loads and gene expression by bats and by *P. destructans*. This approach was recently used to examine the effect of torpor on the transcriptomic response of *M. lucifugus* to WNS [8]. However, samples from intact, lesion-negative wing tissue in that study were treated as “negative” for *P. destructans* (analogous to typical “control,” uninfected samples in most disease ecology studies). In bats hibernating with WNS, wing membrane that does not contain lesions is still covered with measurable quantities of active *P. destructans*, present in the biofilm on the wing surface [18,34,36,41]. Thus, lesion-negative samples are not true “controls” because they do not represent uninfected bats. Instead, the paired-biopsy study design enables a comparison of the host response to the fungus, and fungal response to the host, between scenarios where *P. destructans* is growing in a putative saprophytic state on the wing surface, and a pathogenic state in lesions where fungal flavins are produced [34].

**Figure 1.**
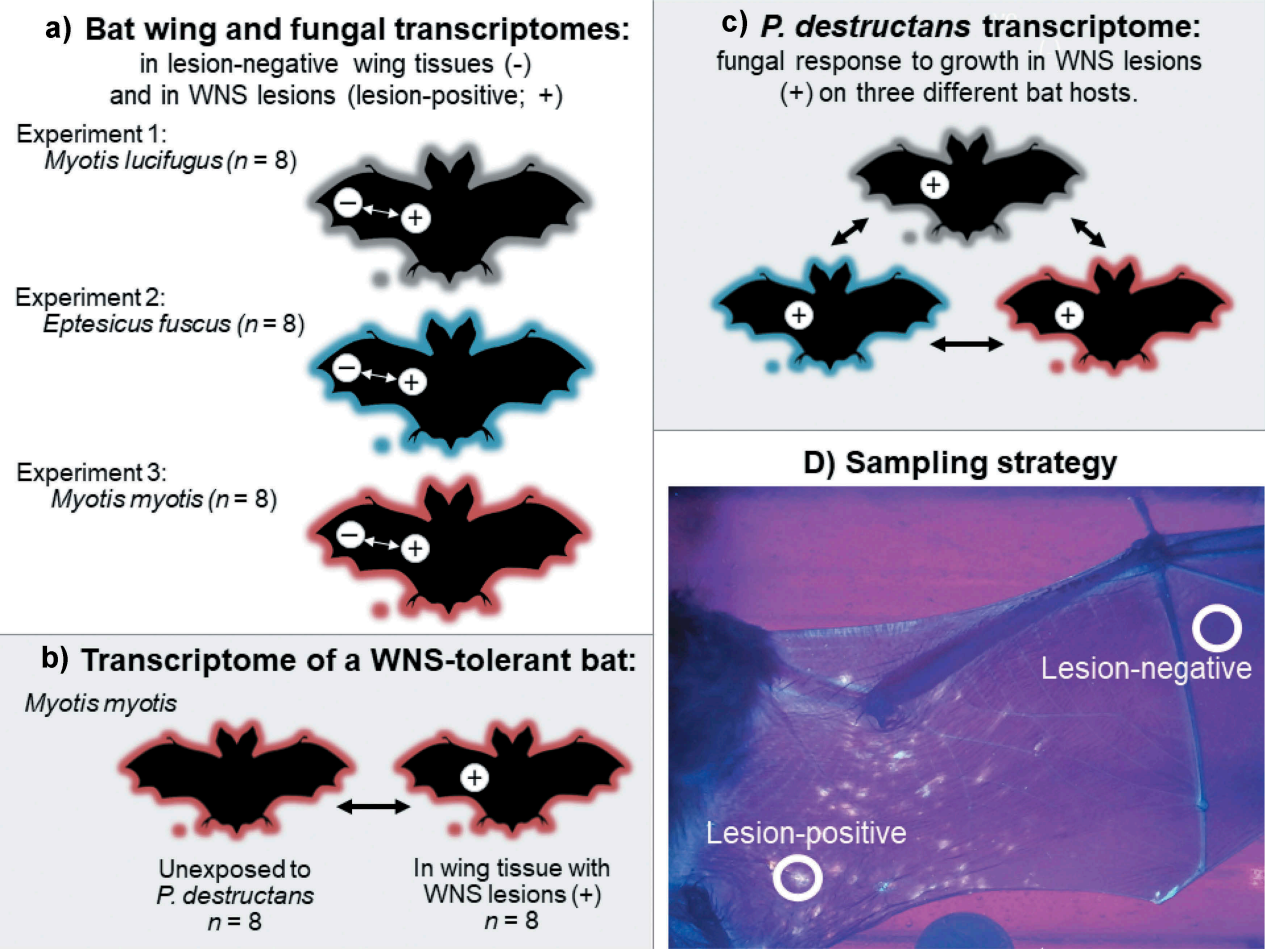
Schematic of comparisons made in this study: (a) three experiments investigating bat and fungal responses to growth in pathogenic and saprophytic contexts. Experimentally infected *Myotis lucifugus* (Experiment 1) and *Eptesicus fuscus* (Experiment 2) hibernated post-exposure under controlled conditions in captivity, and hibernating, wild *Myotis myotis* (Experiment 3) were sampled at the end of hibernation with natural exposure to *Pseudogymnoascus destructans*. (b) characterization of the response to bat white-nose syndrome (WNS) in a tolerant host, (c) comparison of the response of *P. destructans* to growth in a pathogenic context on three host species, based on data collected in (a), and (d) transillumination of the wings of bats exhibiting clinical signs of white-nose syndrome, allowing detection of fluorescing (lesion-positive) and non-fluorescing (lesion-negative) sites for paired-biopsy sampling.

In this study, we conducted three independent experiments, sampling three species of bats that vary in their WNS susceptibility after each had been exposed to *P. destructans* and developed clinical signs of WNS. We quantified and compared the response of each species to saprophytic growth of *P. destructans* on the wing surface, versus pathogenic growth of fungus invading the wing tissue (Figure 1). Naïve populations of Nearctic *M. lucifugus* are highly susceptible to WNS and can exhibit >95% mortality when hibernacula are first exposed to *P. destructans* [1,20,42]. Nearctic *Eptesicus fuscus* may also develop clinical signs when infected with *P. destructans*, but exhibit lower disease severity [20,27,43]. For our first two experiments, we experimentally exposed both species to *P. destructans* and then hibernated them under the same controlled environmental conditions for ~2.5 months. For our third experiment, we collected paired samples from hibernating, free-ranging, naturally infected *M. myotis*. This Palearctic species co-evolved with *P. destructans* [18] but has the highest infection intensity among Palearctic bats [17]. For all species, we hypothesized that *P. destructans* might increase lipase production during saprophytic growth to allow degradation of keratin and sebum in the epidermis, and might increase collagenase production (metalloproteases, destructins, and other serine proteases) during pathogenic growth in lesions. Within each experiment, we contrasted relative fungal loads and transcriptomes of *P. destructans* growing in saprophytic and pathogenic contexts. We qualitatively compared the responses of each bat species to fungal infection, and the response of the fungus to growth in WNS lesions on each species during hibernation. Finally, we compared the wing transcriptome of infected, hibernating *M. myotis* and hibernating *M. myotis* that were not exposed to *P. destructans* [21], to characterize the molecular response of a hibernating, tolerant host species to clinical WNS.

## Methods

In our first two experiments, we collected lesion-positive and lesion-negative wing biopsy samples from torpid *M. lucifugus* and *E. fuscus* hibernating in captivity. These individuals hibernated in the same facility, under controlled conditions, for a similar hibernation period, following experimental exposure to a controlled dose of the same Nearctic isolate of *P. destructans* (Figure 1). The *M. lucifugus* hibernated for 71–73 d post-inoculation and the *E. fuscus* hibernated for 75–77 d before sampling. Both species were sampled while still torpid, to allow the characterization of a “hibernation” transcriptome in the host and pathogen.

In the third experiment, we collected similar, paired-biopsy samples from torpid *M. myotis* hibernating under natural conditions in an abandoned mine in the Jeseniky mountains, Czech Republic. These individuals were naturally exposed to the local Czech strain of *P. destructans* upon entering hibernation (Figure 1). *Myotis myotis* is tolerant of WNS (i.e. it develops only mild clinical signs). Samples were therefore collected after the bats had reached late hibernation to allow fungal load and number of fluorescing WNS lesions to peak before sampling [17, Supporting Information]. Full details of animal collection, care, permits and accession numbers for these samples are provided in the Supporting Information.

We used ultraviolet (UV) transilluminators to visualize fluorescent WNS lesions on the wings of each bat [35], and collected a 4-mm wing biopsy from a part of the wing with no visible fluorescence. These lesion-negative samples contained intact bat skin and superficial fungal growth (*P. destructans* exhibiting putative saprophytic growth; Figure 1). A second 4-mm sterile biopsy from each individual was centered over fluorescing lesions. These lesion-positive samples included bat tissue with cupping erosions and/or deeper dermal invasion [44], containing *P. destructans* exhibiting putative “pathogenic” growth (Figure 1). This approach allowed the collection of samples from the three species that involved a comparable infection grade of invasive fungal growth through living bat skin tissues, which is pathognomonic (diagnostic) for WNS lesions in Nearctic and Palearctic species of bat [18,45]. The lesion-positive samples may have also contained small quantities of fungus growing on the skin surface directly adjacent to lesions [36]; thus, our comparison of the two sample conditions represented a minimum estimate of the differences in fungal load and host or pathogen gene expression between the two contexts.

All biopsy samples were placed immediately in RNAlater (Qiagen) and stored at −80°C prior to RNA sequencing.

### RNA isolation, cDNA library preparation, and RNA-sequencing

Total RNA was isolated using the animal tissue RNA purification kit (Norgen Biotek Corp., Ontario, Canada) with the following modifications to the manufacturer’s protocol. Genomic DNA was removed from the lysate using the gDNA removal column and the purification column from the manufacturer’s single-cell RNA purification kit was substituted to allow RNA elution in a final volume of 15 µL. We confirmed that all samples contained detectable amounts of intact RNA using an RNA pico 6000 chip (Agilent Technologies, California) and resolved the RNA on a 2100 Bioanalyzer (Agilent Technologies, California). We enriched Poly(A)+ RNA using the NEBNext poly(A) mRNA magnetic isolation module (New England Biolabs, Massachusetts), and prepared cDNA libraries using the NEBNext ultra II directional RNA library preparation kit for Illumina (New England Biolabs, Massachusetts). For each species of bat, 16 barcoded libraries were pooled in equimolar quantities and RNA sequencing was performed using 75% of an Illumina NextSeq-500 (Illumina California) run, generating ≥25 million raw paired-end 75 base pair stranded sequences, per library. Sequence quality for each dataset was assessed using FastQC v0.11.7 [46]. We removed Illumina adapter sequences and low-quality bases from all sequences using Trimmomatic v0.36 [47] with the settings ‘ILLUMINACLIP:TruSeq3-PE-2.fa:2:30:10 LEADING:3 TAILING:3 SLIDINGWINDOW:4:15 MINLENGTH:36ʹ.

### Differential gene expression (DGE) analysis for *P. destructans* and bats

Quality-trimmed sequences from all *M. lucifugus* and *E. fuscus* libraries were aligned to their corresponding reference genome using HISAT2 v2.1.0 [48] with the setting “rna-strandness RF.” For *M. lucifugus* we used the ‘Myoluc2.0ʹ genome assembly and gene models from Ensembl 92 [49, accessed April 2018]. For *E. fuscus*, we used the NCBI ‘*Eptesicus fuscus* Annotation Release 100ʹ genome assembly and gene models (accessed April 2018; https://www.ncbi.nlm.nih.gov/genome/annotation_euk/Eptesicus_fuscus/100/). We counted the number of sequences mapping to gene annotations using FeatureCounts v1.6.1 [50], with settings: strand-specific mode (reverse stranded), count paired-end reads as fragments, count only the fragments where both reads aligned successfully, and count multi-mapping fragments.

We generated a single *de novo* transfrag assembly for *M. myotis* using Trinity v2.6.6 [51, setting “strand-specific mode (RF)”]. The assembly included the quality-trimmed sequences from the 16 new *M. myotis* libraries (described above), as well as previously described sequences from *M. myotis* wing samples not exposed to *P. destructans* [Mymy‐Neg1 – Mymy-Neg8, 14b; *n = *8], and *M. myotis* wing samples exposed to *P. destructans* [Mymy‐Pos1 – Mymy-Pos8, 14b; *n = *8]. We used BUSCO v3.0.2 and the “laurasiatheria_odb9 (eukaryota)” dataset to assess the completeness of our *de novo* transfrag assembly [52]. Sequences from each individual *M. myotis* library were aligned to the *de novo* transcript assembly using Bowtie v1.2.2 [53] with the setting “SS_lib_type RF” and transfrag abundance was estimated using RSEM v.1.3.0 [54]. We used RNAmmer v1.2 [55] to identify transfrags representing rRNA sequences, which were removed from the RSEM “count” files before DGE analyses were conducted.

Quality-trimmed sequences from all RNA-seq libraries were aligned to the *P. destructans* reference genome with HISAT2 using the settings described above. For *P. destructans*, we used the ‘ASM164126v1ʹ genome assembly and gene models from Ensembl 92 [49,56, accessed April 2018]. For each library, we used FeatureCounts with the previously described settings to estimate the number of sequences that mapped to a *P. destructans* gene. We used the proportion of transcripts from each sample that aligned to the *P. destructans* genome as a proxy for relative abundance of *P. destructans* in the 48 samples. We used generalized linear mixed effects models (function: glmer, family = binomial; one model for each species) with the lme4 package [57] to estimate the effect of condition (lesion-negative or lesion-positive; fixed effect); on the proportion read alignment to the *P. destructans* genome (response variable). We included individual random effects in each model because there were lesion-positive and lesion-negative samples from the same individuals (paired sampling design), and included an observation-level random effect in each model because the data were overdispersed.

The DGE comparisons of each bat species’ response to lesion-positive and lesion-negative conditions were analyzed independently using the DESeq2 v1.16.1 [58] and edgeR v3.18.1 [59] modules in SARTools v1.6.1 [60]. We used the statistical model “~ individual + condition” for comparisons that contained paired samples from the same individual, using a false discovery rate (FDR) threshold <0.05 [8]. For DESeq2 we used the settings: cooksCutoff = TRUE (perform outliers detection), independentFiltering = TRUE, alpha = 0.05 (threshold of statistical significance), pAdjustMethod = BH (Benjamini/Hochberg p-value adjustment method), typeTrans = rlog (transformation for PCA), and locfunc = median (estimate size factors). For edgeR we used the settings: alpha = 0.05, pAdjustMethod = BH, cpmCutoff = 1 (counts-per-million cutoff), normalizationMethod = TMM (normalization across samples using the trimmed mean of M-values method). These two methods operate under slightly different assumptions and therefore identify a slightly different group of differentially expressed transcripts. In the results, we report transcripts and numbers of transcripts based on the overlap between the two analyses [29], and present independent results from each analysis in the Supporting Information. The *P. destructans “*between-group” (DGE) comparisons replicated the bat “between-group” DGE comparisons using SARTools (i.e., comparing lesion-positive to lesion-negative samples within each species), but with FDR threshold <0.001 [29].

To characterize the response of a tolerant species (*M. myotis*) to WNS, we also performed a DGE comparison between *M. myotis* samples from lesion-positive samples (this study) and wing samples from hibernating individuals not exposed to *P. destructans* [21]. We compared gene expression between these samples using SARTools, where “~condition” was used as the statistical model. We removed “individual” as a factor in this analysis, as this was no longer a paired design. Sequences for *M. myotis* transfrags identified in the DGE analyses were used in NCBI blast 2.5.0+ [61] blastx queries against the *M. lucifugus, P. destructans*, and Swiss-Prot protein databases (accessed June 2018), using an e-value threshold of 10^−5^. We classified transfrags as “bat” or “fungal” transcripts by selecting the blastx hit with the lowest e-value.

We conducted gene ontology (GO-)term enrichment analyses using GOATOOLS v.0.8.4 based on a Fisher’s exact test [62], with a Benjamini/Hochberg FDR correction threshold of p < 0.05 [8]. GOATOOLS requires a list of gene names identified in the DGE analysis, a “population” file that lists the gene names in the genome, and an “association” file that maps a gene name to a GO-term. For GO-term enrichment tests, we included genes that were identified by both the DESeq2 and EdgeR analysis with a two-fold minimum change in transcript level. To generate an “association” file for the *M. lucifugus* GO-term enrichment test, we downloaded a file from the Ensembl 92 BioMart database [63, accessed May 2018] that linked *M. lucifugus* gene names to GO-terms. All 19,728 *M. lucifugus* protein-coding gene names were listed in the *M. lucifugus* “population” file. We modified this strategy for *E. fuscus* and *M. myotis* based on differing available genomic resources. To generate an “association” file for *E. fuscus*, we first used NCBI blast 2.5.0+ blastp with an e-value threshold of 10^−5^ to find *E. fuscus* proteins with sequence similarity to *M. lucifugus* proteins. GO-terms from *M. lucifugus* proteins were linked to their corresponding *E. fuscus* orthologs. To annotate any *E. fuscus* proteins without sequence similarity to *M. lucifugus* proteins, we used NCBI blast 2.5.0+ blastp and the Swiss-Prot reference protein database (accessed June 2018) to identify orthologs, which we queried for associated GO-terms in the UniProt database [64, accessed June 2018]. The results of both searches were combined to create the *E. fuscus* “association” file. All 18,366 *E. fuscus* protein-coding gene names were used in the “population” file. Finally, the large number of transfrags identified in the *M. myotis de novo* transcriptome assembly prohibited the generation of an *M. myotis* “population” file. Therefore, we identified *M. lucifugus* orthologs to the *M. myotis* transfrags detected in both DGE analyses using NCBI blast 2.5.0+ blastx with an e-value threshold of 10^−5^. This *M. lucifugus* ortholog list was substituted for the *M. myotis* transfrags, and we used the *M. lucifugus* “population” file.

A DGE analysis directly comparing gene expression among the three bat species would not have been appropriate because the sequences for each species were processed slightly differently, as described above, and because of the variation in potential confounding factors between the experiments (e.g. different environmental conditions and *P. destructans* strains affecting the captive Nearctic and free-ranging Palearctic bats; Supporting Information). However, we qualitatively explored similarities in the three bat species’ responses to the two contexts (lesion-negative and lesion-positive wing tissue) by quantifying the inter-species overlap in the wing transcriptomes, following 30.

The same caveats about variation in experimental conditions apply to direct comparison of fungal transcriptomes between the three experiments. However, the sequence processing was identical for the fungal transcriptomes, so we were able to conduct a comparison of fungal gene expression within lesions among the three species. Other studies of WNS have conducted a statistical comparison of transcriptome data among different experimental conditions [28,30,37]. We present such an analysis below, based on evidence that Nearctic and Palearctic strains of *P. destructans* produce similar disease outcomes in bats [12,18,44]. Nevertheless, we acknowledge that the results of this analysis must be interpreted carefully given the involvement of a different strain of *P. destructans* in one experiment, and different conditions experienced by the three host species before and during hibernation with WNS.

## Results

We sequenced RNA from paired lesion-negative and lesion-positive samples from hibernating, captive *M. lucifugus* and *E. fuscus*, and from free-ranging, hibernating *M. myotis* (n = 8 sample pairs/species). Illumina sequencing of the 48 resulting libraries produced ∼1.3 billion reads, with an average of 27.9 million reads/library (range: 18.1–72.4 million paired-end (PE) reads/library). Of the remaining ∼1.1 billion trimmed, PE reads, ∼821.6 million aligned to bat genomes, and ∼56.2 million aligned to the *P. destructans* genome (Table S1). Average alignment of “bat reads” to the respective bat genomes was 78.5% for *M. lucifugus*, 72.0% for *E. fuscus*, and 86.4% for *M. myotis*, which we aligned to a *de novo* transcript assembly.

### Fungal growth in saprophytic vs. pathogenic contexts

**Figure 2.**
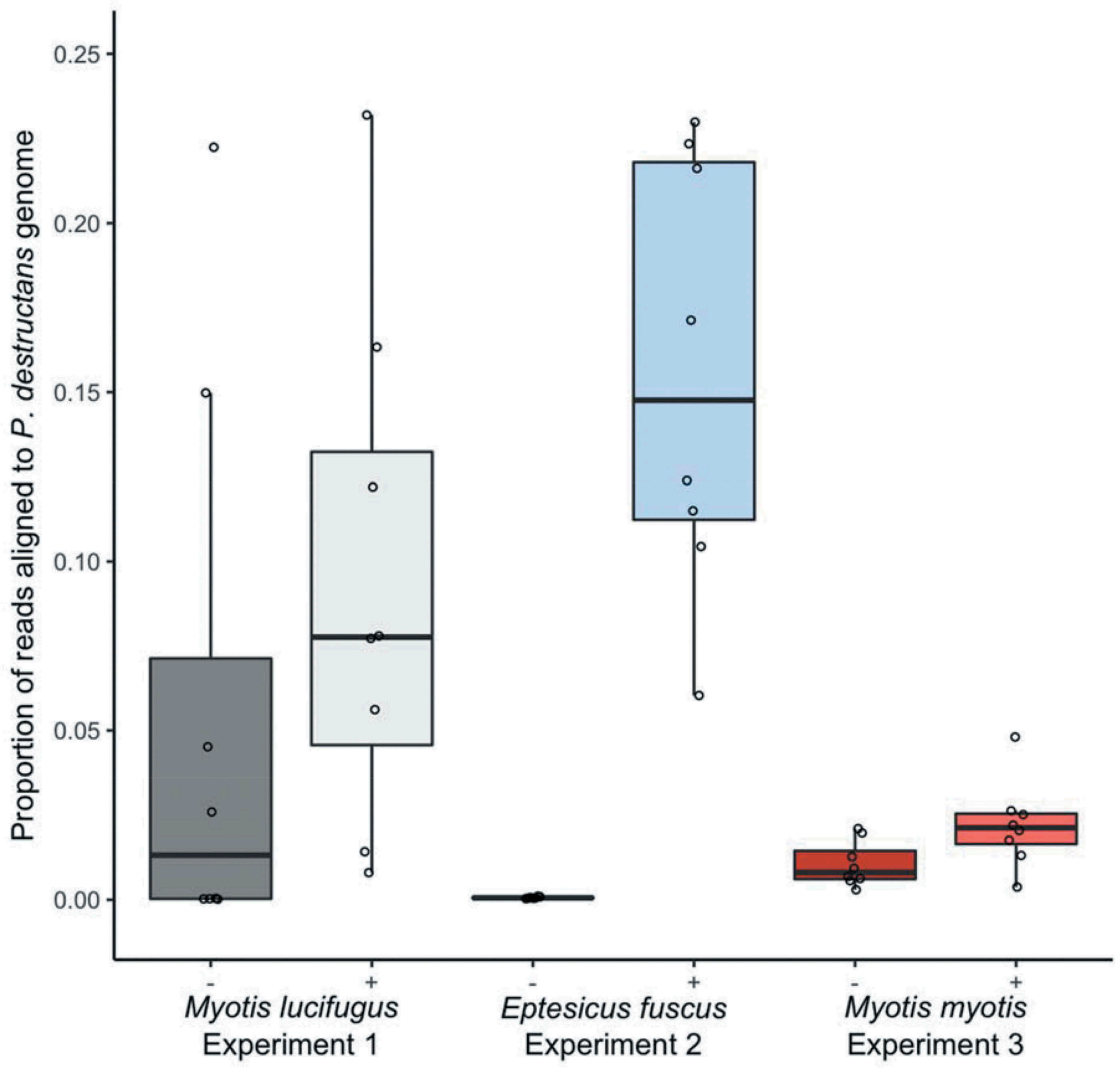
Proportion of paired-end reads from each sample that aligned to the *Pseudogymnoascus destructans* genome in paired lesion-negative (–) and lesion-positive (+) biopsy samples from the wings of bats exhibiting clinical signs of white-nose syndrome (n = 8 pairs of samples per species). *Myotis lucifugus* and *Eptesicus fuscus* were experimentally exposed to a Nearctic isolate of *Pseudogymnoascus destructans* prior to hibernation in captivity under controlled environmental conditions; free-ranging *Myotis myotis* were naturally exposed to a Palearctic strain of *P. destructans* during hibernation in a site in the Czech Republic. Proportion of reads per samples aligning to the *P. destructans* genome provide a proxy for the relative amounts of active *P. destructans* in each sample. Boxplots show median (mid-line) and mean (x).

We used the percent of transcripts from each sample that aligned to the *P. destructans* gene as a proxy for relative abundance of *P. destructans* among the 48 samples. Lesion-positive samples from *E. fuscus* contained the highest proportion of reads aligning to the *P. destructans* genome (mean ± SE: 15.55% ± 2.25%) and the lowest proportion was observed in lesion-negative samples from *E. fuscus* (0.06% ± 0.01%; Figure 2). Lesion-positive samples from all three species contained a higher proportion of reads aligning to the *P. destructans* genome than lesion-negative samples (Figure 2; *M. lucifugus*: 0.056 ± 0.030%, χ^2^ = 5.75, P = 0.016; *E. fuscus*: χ^2^ = 784.19, P < 0.0001; *M. myotis*: χ^2^ = 13.16, P = 0.0003). The difference in the proportion of aligned reads between lesion-positive and lesion-negative was highest in *E. fuscus*, intermediate in *M. lucifugus*, and smallest in *M. myotis* (Figure 2).

### Fungal response to putative saprophytic and pathogenic growth in vivo

We conducted a DGE analysis of the transcriptome of *P. destructans* growing in lesion-positive and lesion-negative wing tissue. For each experiment, we included only paired samples for which both samples contained >50,000 reads aligning to the *P. destructans* genome (8 *M. myotis* and 4 *M. lucifugus)*, thus meeting or exceeding thresholds used in similar studies [8,28,29]. On *M. myotis* wings (*n = *8 bats), only 2 (DESeq2) or 4 (edgeR) uncharacterized genes were differentially expressed. On *M. lucifugus* wings (*n = *4 bats), no genes were differentially expressed by *P. destructans* between the two conditions.

### Fungal responses to pathogenic growth on different host species

**Figure 3.**
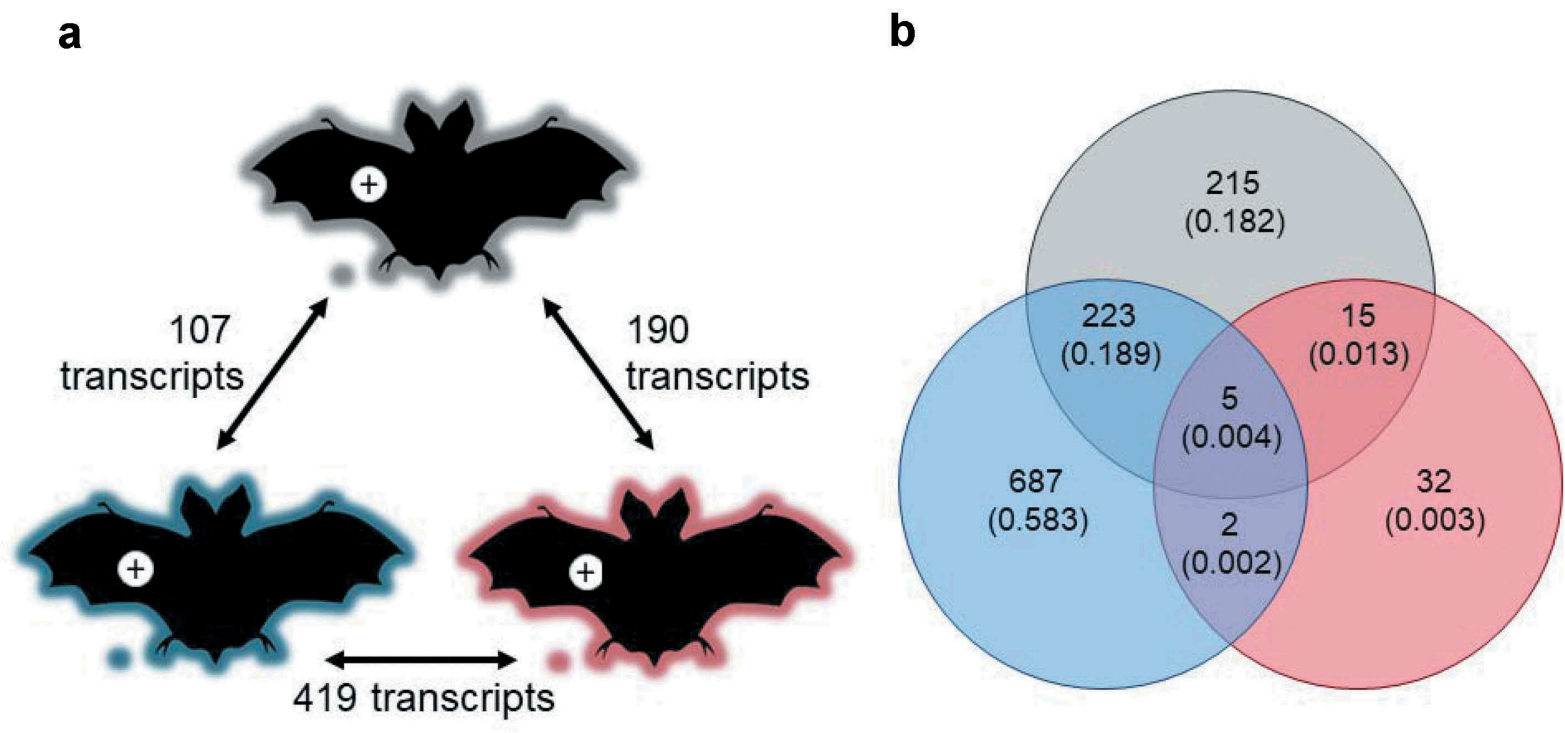
(a) Response of *P. destructans* to growth in WNS lesions on bat wings was most similar between *Myotis lucifugus* (gray) and *Eptesicus fuscus* (blue), and most different between *M. myotis* (red) and *E. fuscus*, but no GO-terms were enriched in the differentially expressed transcripts. Host response to the fungus varied greatly among bat species, shown by (b) low overlap among differentially expressed transcripts identified in each within-species comparison of lesion-negative and lesion-positive tissues, but no GO terms were enriched in the overlapping sets of transcripts.

**Figure 4.**
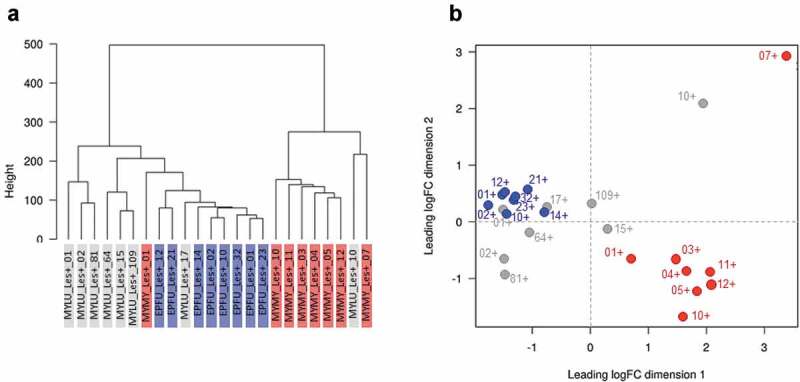
The response of *Pseudogymnoascus destructans* to growth in lesions on bat wings overlapped among three host species, with no consistent, biologically meaningful, interspecific differences in expression of transcripts (no gene ontology categories were enriched). Dendrogram (a) and multi-dimensional scaling plot (b) show lack of clear clustering by host species for *P. destructans* growing on *Myotis lucifugus* (gray-shaded labels on left, gray dots on right); *Eptesicus fuscus* (blue-shaded labels on left; blue dots on right); and *M. myotis* (red-shaded labels on left; red dots on right).

There were pronounced differences in a range of factors among our experiments that could have influenced transcriptomic responses of *P. destructans* (see methods). Despite this variation, however, *P. destructans* responded similarly to growth in lesions on *M. lucifugus, E. fuscus*, and *M. myotis* (*n = *8 per species). We identified 757 differentially expressed transcripts among the three pairwise comparisons (Figure 3, Table S2), but no gene ontology (GO) enrichment. Clustering analyses (dendrograms and multi-dimensional scaling plots) showed that host species (or related, confounding variation among the three experiments) did not explain the variation in the transcriptomic response of *P. destructans* to growth in lesions (Figure 4).

We queried the data for specific, differentially expressed *P. destructans* transcripts highlighted by previous studies [8,28,38,39], and for transcripts related to keratin, sebum, and collagen degradation. Up-regulation of these transcripts varied among species, with no obvious association to host susceptibility (Table 1). Expression of the subtilisin-like serine proteases Destructin-1, −2, and −3 also varied among species, with the greatest expression observed during growth on WNS-tolerant *M. myotis*, and the lowest expression on *E. fuscus*.

**Table 1.** Genes related to putative virulence factors, differentially expressed by *Pseudogymnoascus destructans* during growth in lesions on the wings of *Myotis myotis* (*n = *8; red text), *M. lucifugus* (*n = *8; gray text), and *Eptesicus fuscus* (*n = *8; blue text).

			Differentially expressed^a^ by *P. destructans* during growth on:		
ASM Locus ID	Swiss-Prot BLASTp hit	Description	*M. myotis* vs. *M. lucifugus*	*M. myotis* vs. *E. fuscus*	*E. fuscus* vs. *M. lucifugus*
VC83_00241	PRTA_ASPNG	Aspergillopepsin-2 (Acid protease A; Aspergillopepsin II)		up on *M. myotis*	up on *M. lucifugus*
VC83_06748	Y5950_ASPFU	Aspergillopepsin A-like aspartic endopeptidase		up on *M. myotis*	
VC83_01523	PEPA_ASPOR	Aspartic protease (Aspergillopepsin A/I/O)	up on *M. myotis*	up on *M. myotis*	
VC83_03986	CTSD_TRIVH	Aspartic-type endopeptidase		up on *E. fuscus*	
VC83_01226	LIP1_ARTBC	Secreted lipase		up on *M. myotis*	
VC83_00947	LIP_THELA	Triacylglycerol lipase	up on *M. myotis*	up on *M. myotis*	
VC83_01661	ABD12_XENTR	Monoacylglycerol lipase			
VC83_01744	LPP60_MOUSE	Lysophospholipase		up on *E. fuscus*	up on *E. fuscus*
VC83_01916	SRF1_YEAST	Regulator of phospholipase D SRF1	up on *M. myotis*		
VC83_04822	LIP3_ARTBC	Secreted lipase	up on *M. myotis*		
VC83_08779	PGC1_SCHPO	Phosphatidylglycerol phospholipase C	up on *M. myotis*		
VC83_06062	SUB2_PSED2	Subtilisin-like serine protease (Destructin-1)		up on *M. myotis*	
VC83_04892	SUB1_PSED2	Subtilisin-like serine protease (Destructin-2)		up on *M. myotis*	up on *M. lucifugus*
VC83_09074	SUB3_PSED2	Subtilisin-like serine protease (Destructin-3)	up on *M. myotis*	up on *M. myotis*	
VC83_04862	WSS1_SCHPO	DNA-dependent metalloprotease WSS1 homolog		up on *E. fuscus*	
VC83_04891	MEP1_SORMK	Extracellular metalloprotease		up on *M. myotis*	up on *M. lucifugus*
VC83_02543	PEPS_ASPPH	Carboxypeptidase		up on *M. myotis*	
VC83_05858	UBP34_HUMAN	Ubiquitin carboxyl-terminal hydrolase			up on *E. fuscus*
VC83_08426	OTUB1_HUMAN	Ubiquitin thioesterase			
VC83_02181	SED2_ASPFU	Tripeptidyl-peptidase sed2 (Sedolisin-B)		up on *M. myotis*	up on *M. lucifugus*
VC83_06276	SED2_ASPFU	Tripeptidyl-peptidase sed2 (Sedolisin-B)		up on *M. myotis*	up on *M. lucifugus*
VC83_03247	A1345_ARTBC	Probable extracellular serine carboxypeptidase		up on *M. myotis*	up on *M. lucifugus*
VC83_03453	USTP_ASPFN	Peptidase (Ustiloxin B biosynthesis protein)		up on *E. fuscus*	
VC83_09282	DUG2_YEAST	Di- and tripeptidase		up on *E. fuscus*	

^a^Considered significantly different if fold change >2 and adjusted *P*-value (Benjamini-Hochberg adjustment) <0.001 on both DESeq and edgeR analyses.

### Bat responses to WNS

**Figure 5.**
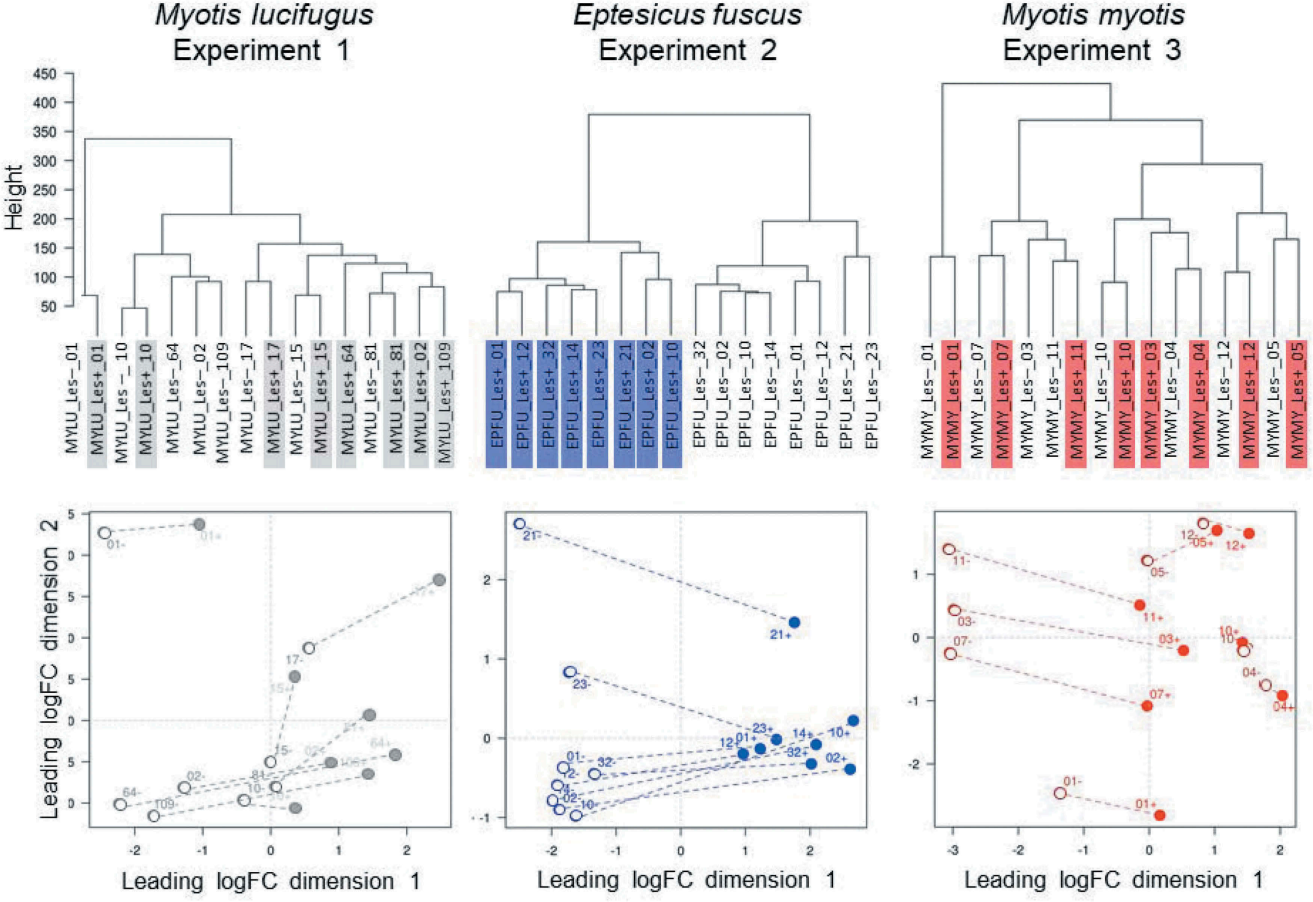
Response of the wing tissue of bats with white-nose syndrome (WNS; *n = *8 pairs of samples/species) to superficial growth of *Pseudogymnoascus destructans* on the wing (lesion-negative, unshaded labels on the dendrograms, above; open circles on the multi-dimensional scaling (MDS) plots, below), compared to growth in a fluorescing WNS lesion on the same individuals (lesion-positive; shaded labels on dendrograms, above; filled circles on the MDS plots, below). Dashed lines on the MDS plots indicate paired lesion-positive (+) and lesion-negative (-) samples from the same individual. *Myotis lucifugus* exhibits an apparent systemic response, with transcriptomes of lesion-negative samples most similar to the paired lesion-positive samples from the same individual. In contrast, *Eptesicus fuscus* mounts a localized response to *P. destructans*; gene expression is more similar between lesion-positive and lesion-negative groups of samples than between paired samples from individual bats. *Myotis myotis*, like its congener, exhibits a systemic response to WNS, but with less intraindividual variation than *M. lucifugus.*

Clustering analyses for paired lesion-negative and lesion-positive samples from *M. lucifugus* suggested a systemic response to WNS (lesion-negative samples were most similar to their paired lesion-positive samples) (Figure 5). We identified 458 differentially expressed transcripts between the two conditions (FDR ≤ 0.05, FC ≥ 2; Table S3). Lesion-positive tissue exhibited up-regulation of genes involved in the regulation of immune system (*ANXA6*), recruitment of effector immune cells to inflammation sites (*CCR1*), regulation of immune/inflammatory response (*IL10, IL1B, IL27A, IL6*), and the innate immune system (*TLR2, TLR4*). However, GOATOOLS analysis found enrichment for only a single GO-term (GO:0016020; CC; membrane; *P = *0.0092).

In contrast, gene expression in the wings of *E. fuscus* with WNS was more similar between lesion-positive and lesion-negative groups of samples than between paired samples from individual bats, suggesting that *E. fuscus* mounts a localized, non-systemic response to *P. destructans* (Figure 5). We identified 939 differentially expressed transcripts between the two conditions (FDR ≤ 0.05, FC ≥ 2). Up-regulated transcripts included several interleukins, chemokines, and protein kinases (Table S4). The GOATOOLS enrichment test identified two categories: GO:0005886 (CC; plasma membrane; *P = *0. 0021), and GO:0003676 (MF; nucleic acid binding; *P = *0.0021).

In *M. myotis*, the transcriptomes of 7/8 individuals also suggested a systemic response (lesion-negative samples were most similar to their paired lesion-positive samples; Figure 5). Only 69 transfrags were differentially expressed between the two conditions Table S5). No GO-terms were enriched in this comparison, indicating that *M. myotis* either mounted a systemic response when infected with *P. destructans* or exhibit a baseline response during hibernation that allows them to tolerate *P. destructans* and WNS (Figure 5).

To evaluate these two potential scenarios, we compared the lesion-positive transcriptomes to previously published wing transcriptomes of hibernating *M. myotis* that were not exposed to *P. destructans* [21]. We identified 10,999 differentially expressed “bat” transfrags in this comparison (FDR) <0.05, fold change (FC) >2). These had sequence similarity to 6,215 *M. lucifugus* genes, and 539 of these transfrags mapped to 345 unique *M. lucifugus* genes that are associated with an immune/defense-related GO-term (Table S6). These data suggest the upregulation of a systemic response to WNS in *M. myotis* rather than tolerance via a constant, baseline response.

When qualitatively comparing the responses of the three bat species to lesion-positive and lesion-negative conditions, we found that only five transcripts were up-regulated in lesion-positive samples by all three species (Figure 3, Table S7). These transcripts had predicted links to keratin production (type II cytoskeletal 7, type I cytoskeletal 19), metalloreductase STEAP1, a bile salt-activated lipase, and Zinc-Alpha-2-Glycoprotein (ZAG). Further 15 transcripts were differentially expressed between conditions by both *M. myotis* and *M. lucifugus*. Two of these were characterized: one related to *ALDH1A1*, the other related to Claudin 10; both up-regulated in lesion-positive tissue. Two further transcripts were up-regulated by both *E. fuscus* and *M. myotis*: one related to a ubiquitin-like protein, and the other associated with the major allergen I polypeptide chain 2. The greatest overlap of differentially expressed transcripts occurred between *E. fuscus* and *M. lucifugus* (223 transcripts, in addition to the five differentially expressed by all three species). However, no GO-term enrichment was detected in any of these inter-specific comparisons indicating that the detected differences in response to WNS lesions among the three species were likely of negligible biological importance.

## Discussion

Our results suggest that the production of virulence factors and increases in active biomass by *P. destructans* are similar among hibernating host species with varying susceptibility to WNS, even between fungal strains and under varying environmental conditions. The two *Myotis* species both mounted systemic responses to WNS, while *E. fuscus* mounted an extremely localized response to infection after hibernation under controlled conditions identical to those experienced by *M. lucifugus*. Estimated relative biomass of fungus in wing tissue was most different between lesion-positive and lesion-negative samples from *E. fuscus*, suggesting that the fungus exhibited a different growth rate superficially on *E. fuscus* than on *Myotis lucifugus* despite hibernation under controlled, similar conditions. This observation supports the potential importance of species’ wing chemistry or microflora in determining susceptibility, but the fungal sequences from lesion-negative *E. fuscus* samples were so scarce that we could not directly compare the fungal transcriptome in the two conditions in this host species. Our study suggests that increased, systemic up-regulation of immune responses is not a catch-all explanation for reduced WNS susceptibility in some species and provides a baseline against which to measure the evolution of reduced WNS susceptibility in Nearctic species such as *M. lucifugus* and *E. fuscus.*

We hypothesized that *P. destructans* would use increased production of lipases to invade the tissue, and then increase the production of collagenases during growth in WNS lesions. Four of the transcripts produced by *P. destructans* in lesions are similar to aspartic proteases secreted by *Aspergillus* that degrade keratin [65], but these were not consistently up-regulated among species (Table 1). Instead, we observed similar responses by the fungus to growth in saprophytic and pathogenic conditions on *M. myotis* and *M. lucifugus*, supporting the “accidental pathogen” hypothesis [32,37].

We also found no evidence for the hypothesis that *P. destructans* might produce different virulence factors among host species [21]. The consistency of gene expression by *P. destructans* among the three species is particularly striking given the unavoidable variation in potentially confounding factors among the three experiments. These included the putative diversity of *P. destructans* strains between the laboratory and natural infections, and unavoidable environmental differences among the sites where we collected *M. lucifugus* and *E. fuscus* in Canada, the laboratory in which they hibernated, and the abandoned mine where we sampled *M. myotis* in the Czech Republic (Supporting Information). Instead of the “transcriptomic cross-talk” observed in some host–pathogen systems [66], the key differences we observed with our comparative dual RNA-seq approach occurred in the response of the three bat species to the two conditions (lesion-negative or lesion-positive wing tissue).

We characterized the molecular response to WNS in hibernating *M. myotis*, a species that has presumably adapted to tolerate WNS through evolution in sympatry with *P. destructans* [18,67]. Importantly, *M. myotis* shows the highest prevalence and intensity of WNS among Palearctic bat species [18,32,44,68,69]. A recent, paired-sample RNAseq study found no meaningful changes in gene expression between lesion-positive and lesion-negative wing tissue in infected, euthermic *M. myotis*, and concluded that *M. myotis* exhibit no immune response to WNS [30]. However, our comparison of gene expression in infected and uninfected wing tissue from torpid *M. myotis* did reveal a strong systemic response to WNS during hibernation, which included immune responses and which differed from the baseline response of uninfected, hibernating *M. myotis.*

A previous study was not able to characterize the molecular response of *M. myotis* to WNS because the bats that were experimentally exposed to *P. destructans* did not develop clinical signs of WNS, and the fungus was barely detectable on the wings [21]. Here, we again noted low biomass of fungus on the wing surface of *M. myotis*, with only 4/8 lesion-negative samples yielding >50,000 fungal reads (Figure 2). Further research should investigate what characteristics of this species’ wings might inhibit saprophytic growth by *P. destructans* [i.e. 70], and how these characteristics might interact with other factors, including immunogenetic variation among individual hosts, microhabitat selection by hosts during hibernation, or variation in pathogenicity among Palearctic strains of *P. destructans* [17,32,71,72].

Our data corroborate previous descriptions of a systemic response to WNS in susceptible *M. lucifugus* [8,73–75]. However, we (and others) characterized these responses in populations that had not yet undergone a selective sweep from WNS. Strong selection may reduce susceptibility to WNS in persisting populations of affected, Nearctic bats where *P. destructans* is now naturalized [2,22,41,76–78], and may also drive shifting transcriptomic responses to infection associated with tolerance or resistance [30].

We also provided the first characterization of molecular response to WNS in a less-susceptible Nearctic species (*E. fuscus*), whose localized response to infection differed markedly from the systemic response of the two *Myotis* species. The apparent lack of thriving, saprophytic fungal growth on the wing surface of *E. fuscus* (Figure 2) is intriguing: among the three species, the growth of *P. destructans* was apparently slowest on the wing surface of *E. fuscus*, and most rapid in lesions on *E. fuscus*. We speculate that either (1) *E. fuscus* do not mount a systemic response to WNS or (2) we observed an apparent systemic response in the two *Myotis* species because they were responding, locally, to increased activity by *P. destructans* on the skin surface, while *E. fuscus* had such low fungal loads in lesion-negative samples that no response was required. Further comparisons with the wing tissue transcriptome of uninfected *E. fuscus* will be required to test these hypotheses.

These results corroborate other reports of inhibited fungal growth on the wings of *E. fuscus. Pseudomonas fluorescens* isolated from the wings of *E. fuscus* can inhibit the growth of *P. destructans in vitro* [79] and may be protective when applied to susceptible bats hibernating with WNS [80,81]. *Pseudomonas* or other microflora on the wings of *E. fuscus* may have slowed the saprophytic growth of *P. destructans* in our experiment.

Our data demonstrate that a comparative approach to characterizing host–pathogen systems such as WNS is essential to understanding variation in disease outcomes among species, although caution must be applied to comparisons of transcriptomes characterized under slightly different conditions. The transcriptomic response of *P. destructans* is surprisingly consistent among contexts, including different host species that upregulate different biological responses to infection. Our paired sampling design allowed us to control for individual variation in responses, and to show that *P. destructans* also responds similarly to saprophytic and pathogenic contexts. In contrast, the response of one bat species to WNS cannot accurately predict the response of others. Further research can clarify whether survival of WNS in susceptible species such as *M. lucifugus* can be explained by altered or initially rare transcriptomic responses to infection, which might be more similar to those exhibited by tolerant species such as *M. myotis* that have evolved in sympatry with *P. destructans*. More broadly, our study highlights the extreme context-dependence of host–pathogen interactions, reinforcing the need to explicitly consider inter- and intraspecific variation in responses to infectious diseases.

## Supplementary Material

Supplemental Material

## Data Accessibility

RNA-sequencing files will be made available from the NCBI sequence read archive database (accession SRP231237) following manuscript acceptance.
