# Supplementary material for: Transcriptional host–pathogen responses of Pseudogymnoascus destructans and three species of bats with white-nose syndrome
Source: Virulence. 2020 Jun 17;11(1):781–94. doi: 10.1080/21505594.2020.1768018 (PMC7549942; doi:10.1080/21505594.2020.1768018)
Supplement: Supplemental Material [file KVIR_A_1768018_SM0933.zip › Supporting_Information_for_Davy_Donaldson_et_al._2020_Virulence.pdf]

Supporting Information for:

Davy, C.M.*, M.E. Donaldson*, H. Bandouchova, A.M. Breit, N.A.S. Dorville, Y.A. Dzal, V. Kovacova, E.L. Kunkel, N. Martínková, K.J.O. Norquay, J.E. Paterson, J. Zukal, J. Pikula, C.K.R. Willis, and C.J. Kyle. (2020). Transcriptional host-pathogen responses of Pseudogymnoascus destructans and three species of bats during white-nose syndrome. *Virulence*.

This document contains the following information:

1. Collection and care of bats sampled in this study,
2. An ethics statement regarding the use of an endangered bat (*Myotis lucifugus*) in our study, and the permits authorizing this work

All Supplemental Tables (Tables S1 – S7) are in the supplemental .xlsx file, along with the list of sample IDs for this study and their respective SRA accession numbers.

1. Animal care and sample collection

Myotis lucifugus and Eptesicus fuscus

Wing biopsies of *Myotis lucifugus* and *Eptesicus fuscus* for this study were collected from bats that were part of several concurrent laboratory studies on experimentally infected bats at the University of Winnipeg. On 8 January 2017, we collected 67 *M. lucifugus* from Okaw Cave, a *P. destructans*-free hibernaculum about 75 km east of The Pas, Manitoba, Canada (53.8255°N, 101.2476°W). We tested 50 of these *M. lucifugus* for *P. destructans* prior to experimental exposure, as well as a substrate swab sample from the hibernaculum. None of these samples tested positive. The winter started on 14 October 2016, with 86 days until collection and transport to Winnipeg. The mean annual surface temperature at the site is 0°C, resulting in an estimated range of -1.6 to 5.7°C available to hibernating bats. On 18 January 2017, we collected 32 *E. fuscus* from Richard Lake Mine, a hibernaculum about 100 km east of Kenora, Ontario, Canada (49.7670°N; 94.4894°W). Here, the winter started on 22 November, 2016, bringing the

estimated maximum duration until collection and transport to the lab to 57 days. The mean annual surface temperature at the site is 2.3°C, which means that bats can find hibernation roosts in an estimated temperature range of about -0.2 to 7.2°C. *Pseudogymnoascus destructans* was detected in Richard Lake Mine for the first time during winter 2017. Four of eight swabs from *E. fuscus* used in this study tested positive for *P. destructans* (quantitative polymerase chain reaction (qPCR) Ct values <40; Table S1), although the low levels of *P. destructans* in those four samples were near the detection limit of the assay (range = 39.996 – 39.052; mean ± s.d. = 39.495 ± 0.373). No *E. fuscus* had clinical signs of WNS at the time of capture, and there was not yet evidence of WNS in the mine; these bats were experiencing their first winter of hibernation in a site affected by the pathogen.

We collected bats from the walls and ceiling of the hibernacula, and then housed them in individual cloth bags hung within a cooler lined with wet towels to maintain humidity and reduce disturbance. Bats were then transported 30 min by helicopter and 4 h by car from Okaw Cave, and 3 h by car from Richard Lake Mine, to the Animal Facility at the University of Winnipeg. Once the *M. lucifugus* and *E. fuscus* arrived at the University of Winnipeg we obtained morphometric measurements and swabbed the flight membranes for *P. destructans* in a biosafety cabinet using a standardized protocol (Langwig et al. 2015), to confirm infection status via qPCR (see below). We randomly assigned individuals of each species to treatment or control groups. Treatment bats were infected with *P. destructans* collected in 2016 from the Glebe Mine, Waterford County, New Brunswick (Vanderwolf, McAlpine, Forbes, & Malloch, 2012). Inoculum was prepared by pipetting 20 μl of inoculum, containing 500,000 *P. destructans* conidia, suspended in phosphate buffered saline with Tween20 to prevent clumping, onto the wing and tail membranes. Control bats received 20 ul of the identical solution but without fungal

conidia. Only treatment bats were involved in the current study, but we are reporting the entire process here for clarity.

Bats were housed in mesh cages (Exo-terra Flexarium©; 22 cm x 35 cm x 43 cm) in two temperature/humidity-controlled incubators (Caron© Environmental Chamber model 6041; 90.1 cm x 84.5 cm x 228.9 cm). Cages contained bats of the same species and treatment group (n = 8 *E. fuscus* per cage and 16 *M. lucifugus* per cage), with one treatment cage and one control cage per species in each incubator, for a total of eight cages. Both incubators were set to 8 °C and 98% relative humidity to simulate a natural hibernation environment. Water was provided *ad libitum;* food was not provided during hibernation. We monitored cages using motion-activated infrared video (Digital Watchdog© VMAX series) on a weatherproof, dome camera (Speco© technologies, model HD5941T) mounted at the top of each cage to reduce the need to open incubators and disturb bats.

Bats hibernated in captivity at the University of Winnipeg until clinical signs were well established, and lesion-positive and lesion-negative wing samples were collected. We began to collect samples starting 21 March 2017; 71-73 days post-inoculation for *M. lucifugus* and 75-77 days post-inoculation for *E. fuscus* (157 and 132 days since the beginning of winter at the site of origin, respectively). We removed bats from cages and, in the biosafety cabinet, swabbed the flight membranes as described above for subsequent qPCR analysis. We then used ultraviolet (UV) fluorescence to identify lesion-positive and lesion negative regions of wing tissue and collected tissue biopsies (see Methods in the main text).

We tested swabs of flight membranes collected before and after the experiment for the presence of *P. destructans* DNA at the Pathogens and Microbiome Institute at Northern Arizona University, using real-time and standard TaqMan assay qPCR (Bernard et al. 2017; Janicki et al.

2015; Langwig et al. 2013; Muller et al. 2013). Following these earlier studies, we considered any sample with a Ct value below 40 to be positive for *P. destructans*.

Table S1. Real-time polymerase chain reaction (qPCR) results for wing swabs taken from eight big brown bat (*Eptesicus fuscus*) following collection from a hibernaculum. Each sample was tested twice (Assay 1 and Assay 2). Bats were tested prior to experimental inoculation with *Pseudogymnoascus destructans* and hibernation in captivity under controlled environmental conditions. Detection limit of the assay was Ct=40; values >40 were considered negative for *P. destructans.*

Bat ID #	Assay 1 Ct value	Assay 2 Ct value
IE_2017_EPFU_01	negative	negative
IE_2017_EPFU_02	negative	negative
IE_2017_EPFU_10	39.052	39.996
IE_2017_EPFU_12	negative	negative
IE_2017_EPFU_14	39.39	39.25
IE_2017_EPFU_21	negative	negative
IE_2017_EPFU_23	39.662	39.068
IE_2017_EPFU_32	39.989	39.551

Myotis myotis

We collected samples from *Myotis myotis* hibernating under natural conditions in an abandoned mine in the Jeseniky mountains, Czech Republic on 6 May 2016. *Pseudogymnoascus destructans* is endemic in the Czech Republic. At this site (the Šimon and Juda mine; 50.05°N, 17.30°E), the majority of hibernating bats is exposed to *P. destructans*, develops skin infection and clinical signs of WNS when the infection intensity exceeds about 300 skin lesions (Bandouchova et al., 2018). We sampled bats near the end of natural hibernation to allow fungal

load and number of UV-fluorescent WNS lesions to peak before sampling, following data from Martínková *et al.* (2018) (Figure S1). Torpid bats were collected from the walls and sampled non-lethally within < 30 minutes of disturbance (i.e., in the early stages of arousal from hibernation torpor), and then released. To estimate the duration of the hibernation period, we calculated the local start of winter based on daily temperatures and precipitation (Mayes Boustead, Hilberg, Shulski, & Hubbard, 2015). Using data from the nearest meteorological station at Lysá hora, we calculated the start of winter as the first day when maximum daily temperature was ≤ 0°C, daily snowfall was ≥ 0.25cm or the date was at least 1 December. Winter 2015/2016 in the Jeseniky Mts. began on 22 October 2015, bringing the likely maximum duration of hibernation (start of winter until sampling) to about 197 days. The mean annual surface temperature at the site is 4.8^{o}C, and we used a fuzzy regression model (Martínková et al., 2018) to approximate the range of temperatures (1.4 – 8.8^{o}C) available to hibernating bats at the site.

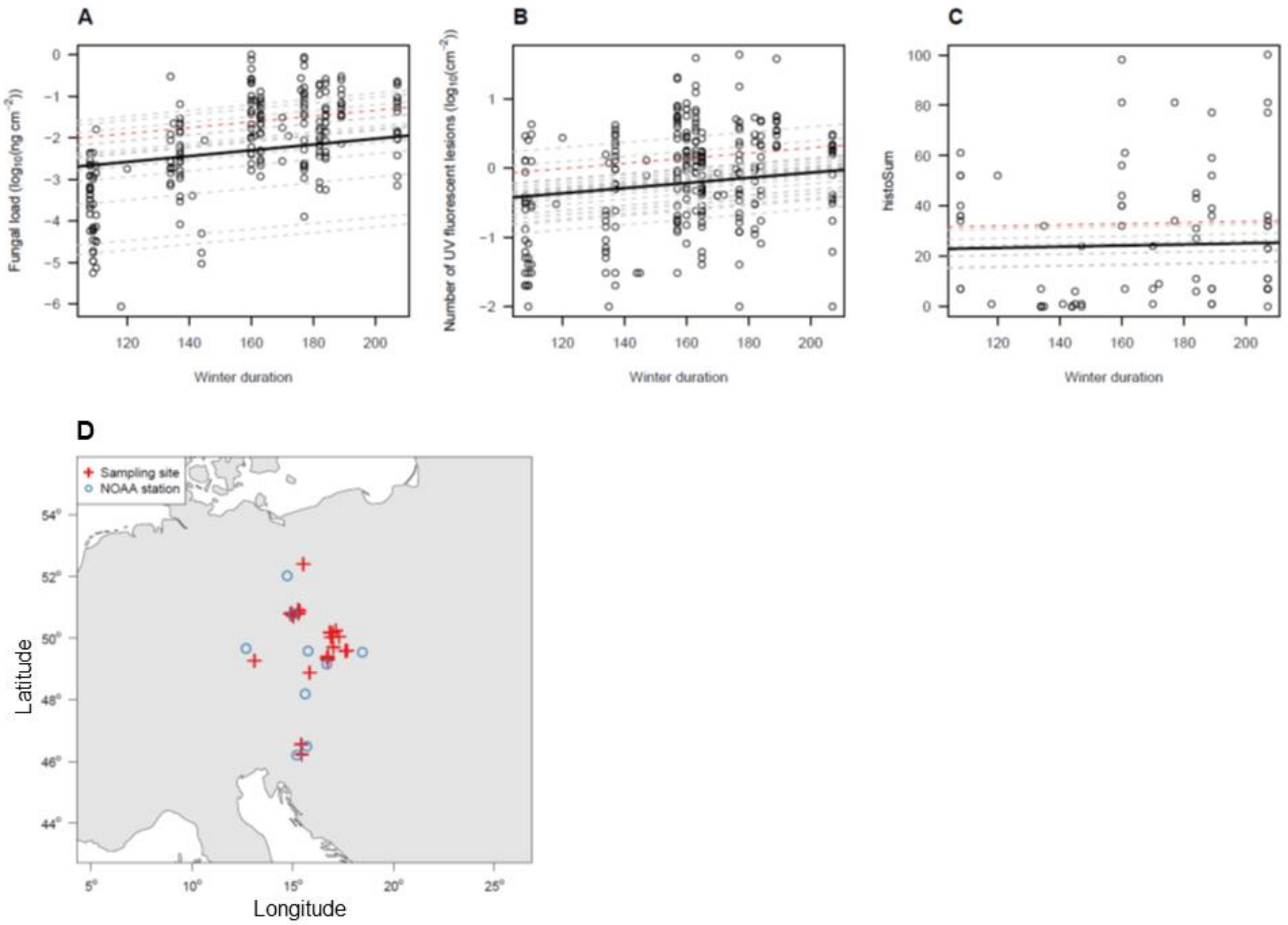

Figure S1: Temporal changes in infection intensity and disease severity in *Myotis myotis* showing that clinical signs relevant to this study peak in early spring prior to emergence from hibernation, based on previously published data (Martínková et al., 2018; Pikula et al., 2017). Winter duration is the number of days since the beginning of winter as per the AWSSI calculation until the day of sampling. Thick line indicates the fixed effects of a GLMM model, dashed lines are random effects for site. The red line shows the random effect for the Simon and Juda mine. A) Quantification of *Pseudogymnoascus destructans* DNA in a wing swab, B) number of orange-yellow fluorescing spots indicative for WNS lesions, and C) WNS pathology score from a wing biopsy. D) Map showing sampling sites in the Czech Republic and the respective meteorological stations that had daily summary data available for the given time period.

2. Ethics statement

Myotis lucifugus is listed as endangered by the International Union for the Conservation of Nature (Solari, 2018). It is listed as endangered at the State level in several of the eastern United States, though not federally, and is listed as endangered in Canada under the Canadian Species At Risk Act (COSEWIC, 2013). *Eptesicus fuscus* is not listed in either country, but populations of *E. fuscus* are also under pressure from WNS (Mcalpine et al., 2016) and from other sources of mortality (Zimmerling & Francis, 2016). Therefore, this study only involved *M. lucifugus* and *E. fuscus* collected from populations that were naïve to WNS, but were expected to soon experience WNS during natural hibernation as the distribution of the pathogen continued to increase. *Myotis myotis* are not considered at risk, but all individuals were released at their capture site after sampling.

Fieldwork and bat sampling in the Czech Republic were performed in accordance with Czech Law No. 114/1992 on Nature and Landscape Protection, based on permits 1662/MK/2012S/00775/MK/2012, 866/JS/2012 and 00356/KK/2008/AOPK issued by the Agency for Nature Conservation and Landscape Protection of the Czech Republic. Free-living bats were handled in accordance with Czech Certificate of Professional Competence No. CZ01341 (§17, Act No. 246/1992).

All work at the University of Winnipeg was approved under the Manitoba Sustainable Development Wildlife Scientific Permit No. SAR16009, the Ontario Ministry of Natural Resources Wildlife Scientific Collector's Authorization 1085301 and University of Winnipeg Animal Care Protocol #AE08399.

References

Bandouchova, H., Bartonička, T., Berkova, H., Brichta, J., Kokurewicz, T., Kovacova, V., … Zukal, J. (2018). Alterations in the health of hibernating bats under pathogen pressure. *Scientific Reports*, *8*(1), 1–11. https://doi.org/10.1038/s41598-018-24461-5

COSEWIC. (2013). COSEWIC assessment and status report on the Little Brown Myotis Myotis lucifugus, Northern Myotis Myotis septentrionalis and Tri-colored Bat Perimyotis subflavus in Canada. (Committee on the Status of Endangered Wildlife in Canada, Ed.). Ottawa. Retrieved from file:///C:/Users/Michael/SkyDrive/CJK Lab/Grants and Accounting/SARRFO 2015 - Pd/Cosewic Assessment and Status Report (2013) - 3-bats endangered.pdf

Martínková, N., Pikula, J., Zukal, J., Kovacova, V., Bandouchova, H., Bartonicka, T., … Zahradníková, A. (2018). Hibernation temperature-dependent Pseudogymnoascus destructans infection intensity in Palearctic bats. *Virulence*, *9*(1), 1734–1750. https://doi.org/10.1080/21505594.2018.1548685

Mayes Boustead, B. E., Hilberg, S. D., Shulski, M. D., & Hubbard, K. G. (2015). The Accumulated Winter Season Severity Index (AWSSI). *Journal of Applied Meteorology and Climatology*, *54*(8), 1693–1712. https://doi.org/10.1175/JAMC-D-14-0217.1

Mcalpine, D. F., Mcburney, S., Sabine, M., Vanderwolf, K. J., Park, A., & Cai, H. Y. (2016). Molecular Detection of Pseudogymnoascus destructans (Ascomycota: Pseudeurotiaceae) and Unidentified Fungal Dermatitides on Big Brown Bats (Eptesicus fuscus) Overwintering inside Buildings in Canada. *Journal of Wildlife Diseases Wildlife Disease Association*, *52*(4), 0–0. https://doi.org/10.7589/2015-03-076

Pikula, J., Amelon, S. K., Bandouchova, H., Bartonička, T., Berkova, H., Brichta, J., … Martínková, N. (2017). White-nose syndrome pathology grading in nearctic and palearctic bats. *PLoS ONE*, *12*(8), 1–21. https://doi.org/10.1371/journal.pone.0180435

Solari, M. (2018). Myotis lucifugus. https://doi.org/http://dx.doi.org/10.2305/IUCN.UK.2018-2.RLTS.T14176A22056344.en.

Vanderwolf, K. J., McAlpine, D. F., Forbes, G. J., & Malloch, D. (2012). Bat populations and cave microclimate prior to and at the outbreak of white-nose syndrome in New Brunswick. *Canadian Field-Naturalist*, *126*(2), 125–134. https://doi.org/10.22621/cfn.v126i2.1327

Zimmerling, J. R., & Francis, C. M. (2016). Bat mortality due to wind turbines in Canada. *The Journal of Wildlife Management*, *80*(8), 1360–1369. https://doi.org/10.1002/jwmg.21128
